# A personalised intervention programme aimed at improving adherence to oral antidiabetic and/or antihypertensive medication in people with type 2 diabetes mellitus, the INTENSE study: study protocol for a randomised controlled trial

**DOI:** 10.1186/s13063-022-06491-7

**Published:** 2022-09-02

**Authors:** Marlous Langendoen-Gort, Hiyam Al-Jabr, Jacqueline G. Hugtenburg, Femke Rutters, Maartje de Wit, Debi Bhattacharya, Ameen Abu-Hanna, Andrew Farmer, Petra J. M. Elders

**Affiliations:** 1 Amsterdam UMC location Vrije Universiteit Amsterdam, Department of General Practice, Boelelaan 1117, Amsterdam, The Netherlands; 2 Amsterdam Public Health Research Institute, Amsterdam, The Netherlands; 3 Integrated Care Academy, University of Suffolk, Ipswich, UK; 4 Amsterdam UMC location Vrije Universiteit Amsterdam, Department of Clinical Pharmacology and Pharmacy, Boelelaan 1117, Amsterdam, The Netherlands; 5 Amsterdam UMC location Vrije Universiteit Amsterdam, Department of Epidemiology and Data Science, Boelelaan 1117, Amsterdam, The Netherlands; 6 Amsterdam UMC location Vrije Universiteit Amsterdam, Department of Medical Psychology, Boelelaan 1117, Amsterdam, The Netherlands; 7 School of Allied Health Professions, University of Leicester, United Kingdom School of Pharmacy, University of East Anglia, Norwich, UK; 8 Amsterdam UMC location University of Amsterdam, Department of Medical Informatics, Meibergdreef 9, Amsterdam, The Netherlands; 9 Nuffield Department of Primary Care Health Sciences, University of Oxford, Oxford, UK

**Keywords:** Type 2 diabetes mellitus, Medication adherence, Personalised intervention programme, Pharmacy

## Abstract

**Background:**

Medication non-adherence is a prevalent health problem in people with type 2 diabetes mellitus (T2DM). Interventions have previously been developed to improve medication adherence, but inconsistent outcomes have been reported. A potential explanation for this inconsistency is a ‘one size fits all’ approach, with interventions not tailored to the needs and preferences of individuals. Therefore, the aim of this study is to evaluate the effectiveness of a personalised intervention programme aimed at improving adherence to oral antidiabetic and/or antihypertensive medication in people with T2DM.

**Methods:**

A parallel-group randomised controlled trial will be conducted in 40–50 community pharmacies in the Netherlands and the United Kingdom (UK). A total of 300 participants will be included and followed up for a period of 6 months. Participants will be people with T2DM identified as non-adherent to oral antidiabetic and/or antihypertensive medication, aged 35–75 years and mobile phone users. The intervention group will receive a personalised intervention programme that is based on one or more of the participants’ pre-defined non-adherence profile(s), namely (I) Knowledge and perceptions, (II) Practical problems, (III) Side effects and (IV) Negative mood and beliefs. The intervention comprises of one or more supporting modules, namely (I) Brief messaging, (II) Clinical medication review, (III) Medication schedule, (IV) Reminding messaging, (V) Medication dispensing systems, (VI) Smart messaging, (VII) Referral to general practitioner and (VIII) Unguided web-based Self Help Application for low mood. The control group will receive usual care including access to a publicly available informative diabetes website. The primary study outcome is medication adherence measured with a telephone pill count. Secondary outcomes are systolic blood pressure, HbA1c level, self-reported medication adherence, attitude and beliefs toward medication, satisfaction with diabetes treatment, health status and medical consumption and productivity cost. In addition, a process evaluation will be undertaken to establish the fidelity, reach and the extent to which intervention delivery is normalised in the daily practice of community pharmacy teams.

**Discussion:**

The study can lead to a personalised intervention programme that improves medication adherence in people with T2DM that are non-adherent to oral antidiabetic and/or antihypertensive medication.

**Trial registration:**

Dutch Trial Register, Trial NL8747, registered 02 July, 2020; ISRCTN Registry, ISRCTN36009809, registered 05 February, 2020.

**Supplementary Information:**

The online version contains supplementary material available at 10.1186/s13063-022-06491-7.

## Administrative information

Note: the number in curly brackets in this protocol refer to SPIRIT checklist item numbers. The order of the items has been modified to group similar items (see http://www.equator-network.org/reporting-guidelines/spirit-2013-statement-defining-standard-protocol-items-for-clinical-trials/).

**Table:** 

**Title {1}**	A personalised intervention programme aimed at improving adherence to oral antidiabetic and/or antihypertensive medication in people with type 2 diabetes mellitus, the INTENSE study: study protocol for a randomised controlled trial
**Trial registration {2a and 2b}**	Dutch Trial Register, Trial NL8747, registered 02 July 2020, https://www.trialregister.nl/trial/8747 ISRCTN Registry, ISRCTN36009809, registered 05 February 2020, https://www.isrctn.com/ISRCTN36009809
**Protocol version {3}**	Article Trials study protocol, version 2, 10 June 2022
**Funding {4}**	The study was funded through an award of the European Foundation for the study of Diabetes (EFSD) supported by Servier
**Author details {5a}**	^1^ Amsterdam UMC location Vrije Universiteit Amsterdam, Department of General Practice, Boelelaan 1117, Amsterdam, The Netherlands ^2^ Amsterdam Public Health research institute, Amsterdam, The Netherlands ^3^ Integrated Care Academy, University of Suffolk, Ipswich, UK ^4^ Amsterdam UMC location Vrije Universiteit Amsterdam, Department of Clinical Pharmacology and Pharmacy, Boelelaan 1117, Amsterdam, The Netherlands ^5^ Amsterdam UMC location Vrije Universiteit Amsterdam, Department of Epidemiology and Data Science, Boelelaan 1117, Amsterdam, The Netherlands ^6^ Amsterdam UMC location Vrije Universiteit Amsterdam, Department of Medical Psychology, Boelelaan 1117, Amsterdam, The Netherlands ^7^ School of Allied Health Professions, University of Leicester, United Kingdom School of Pharmacy, University of East Anglia, Norwich, UK ^8^ Amsterdam UMC location University of Amsterdam, Department of Medical Informatics, Meibergdreef 9, Amsterdam, the Netherlands ^9^ Nuffield Department of Primary Care Health Sciences, University of Oxford, Oxford, UK
**Name and contact information for the trial sponsor {5b}**	Department of General Practice Amsterdam University Medical Centers, Amsterdam PO box 7057 1007 MB Amsterdam, the Netherlands Tel: +31 20-4448199 Email: hag-research@amsterdamumc.nl
**Role of Sponsor**	After having been awarded this study, the sponsor takes responsibility in the collection, analysis, and interpretation of data, and in writing the manuscript.

## Introduction

### Background and rationale {6a}

The International Diabetes Federation has stated that the worldwide prevalence of type 2 diabetes mellitus (T2DM) continues to increase [1]. In 2019, around 463 million adults had diabetes mellitus and it is estimated that this number will increase to 700 million by 2045, of which the majority is affected by T2DM [1].

People with T2DM are initially managed with lifestyle recommendations, aimed at targeting risk factors such as physical inactivity and an unhealthy diet [2]. When lifestyle recommendations insufficiently manage T2DM, antidiabetic medication including for some insulin is prescribed. This is often combined with medication targeting cardiovascular disease risk factors [2]. T2DM treatment is highly effective at regulating blood glucose levels and reducing the risk of developing long-term complications, such as retinopathy, nephropathy, cardiovascular disease and peripheral vascular disease [2].

Between 39 and 93% of the people with T2DM adhere to their prescribed oral antidiabetic medication [3, 4] and 77 and 79% to their antihypertensive medication [5, 6]. This suboptimal adherence causes a considerable group of people with T2DM to have limited glycaemic [7] and cardiovascular control [8]. Consequently, non-adherence increases hospitalisations [8–10], mortality rates [8, 9] and healthcare costs [10, 11].

‘Medication adherence is a complex and multidimensional problem’ [12, 13]. Non-adherence can be classified as intentional and unintentional [14, 15]. Intentional non-adherence is an active and rational decision making process in which patients consciously adjust their medication schedule [14, 15], for instance caused by experiencing side effects. Unintentional non-adherence is a passive process and non-rational behaviour, such as forgetting to take medication [14, 15]. Determinants of non-adherence can be categorised as related to patient, condition, treatment, social/economic and/or health care [16].

In the past decades, several interventions have been developed that aim to improve adherence to antidiabetic medication in people with T2DM [17]. It has been shown that multifaceted interventions that target multiple non-adherence factors are more effective than interventions that only use one strategy [17]. However, the results of similar interventions appear to be limited and inconsistent [17].

This limited effect is comparable to the results of adherence interventions in other patient populations [12, 18–23]. Potential explanations for these inconsistent and limited effects can be that researchers did not measure medication adherence using standardised methods [17], interventions were offered to all patients regardless of whether they were non-adherent [12, 13] and interventions were not tailored to the individual needs of patients [12, 17].

### Objectives {7}

Therefore, the aim of the ‘ImproviNg Treatment adhErence iN people with diabeteS mEllitus’ (INTENSE) study is to evaluate the effectiveness of a personalised intervention programme aimed at improving adherence to oral antidiabetic and/or antihypertensive medication in people with T2DM. The intervention programme will be tailored to a participant’s specific situation, needs and preferences and will only be offered to people that have been identified as non-adherent. The effectiveness of the intervention will be tested in a randomised controlled trial in community pharmacies in the Netherlands and the United Kingdom (UK).

### Trial design {8}

A parallel-group (1:1) randomised controlled trial with a superiority design will be performed in community pharmacies in the Netherlands and the UK. Since participants who receive the intervention condition are encouraged to participate in the selection of the intervention modules, the study has an open design. Participants allocated to the control condition will receive predominantly usual care.

## Methods: participants, interventions and outcomes

### Study design {9}

The study will be carried out in the Netherlands and the UK. In the Netherlands, participants will be identified and recruited in community pharmacies. In the UK, participants will be identified in general practices and recruited in community pharmacies.

### Eligibility criteria {10}

Participants that will be included in the study are people with T2DM identified as being non-adherent to oral antidiabetic and/or antihypertensive medication, aged 35–75 years, mobile phone user and should be able to understand text messages in either Dutch for participants recruited in the Netherlands and English for participants recruited in the UK. Subsequently, participants that are excluded from participation in the study are people that use insulin, have invalid medication adherence data (e.g. due to hospital admittance), are already using medication-intake supporting devices provided by the pharmacy or are suffering from severe mental illness as indicated by self-report.

Eligible participants will be selected with an in-practice electronic search in the dispensing records of participating pharmacies by pharmacists in the Netherlands and in the prescribing records of participating general practices by a member of the general practice team in the UK. A dispensing score or prescribing score of less than 80% is considered non-adherent, which is a commonly used cut-off value [3, 4, 13, 24]. In the Netherlands, the dispensing score will be calculated over a period of 1 year and in the UK the prescription score will be calculated over 2 years.

In the Netherlands, participants will be asked not to participate in any other medical scientific research during participation in the INTENSE study. In the UK, participants that are involved in current research or that have participated in research in the previous 12 months will be excluded from the study.

### Who will take informed consent? {26a}

Potentially eligible patients will receive an invitation letter and patient information leaflet via post. In the Netherlands, this will be posted by participating pharmacy teams and in the UK by participating general practice teams. Potential participants will express their interest by contacting the researchers in the Netherlands and in the UK by contacting the pharmacy teams. In the UK, definitive screening and written informed consent will be obtained by an appropriately trained member of the pharmacy team. In the Netherlands, this will be undertaken by a member of the research team.

### Additional consent provision for the collection and use of participant data and biological specimens {26b}

Participants who are randomised in the intervention group will be asked whether they are willing to have their consultation with the pharmacist audiotaped and whether they may be contacted for an additional interview for the process analysis of the study. There will be no additional consent provisions for biological specimens since these are not collected.

## Interventions

### Explanation for the choice of comparators {6b}

#### Classification of non-adherence

For a previous project on non-adherence, literature was systematically reviewed on the causes of non-adherence and the available interventions to improve adherence [12]. This review showed that there is a wide diversity of non-adherence-related factors. Although several interventions have been shown to reduce non-adherence, even the most effective interventions only had minimal effects. Their lack of effectiveness can be explained by two factors. First, most interventions were not tailored to the needs and preferences of individual patients. Second, most interventions were offered to all patients regardless of whether they were adherent or not. Therefore, a successful intervention should be offered only to non-adherent patients and should be tailored to the specific needs and preferences of the individual patient.

As a follow-up of the review, a semi-structured interview guide was developed, the so-called Quick Barrier Scan (QBS), to assess individual medication non-adherence-related factors and to develop main profiles of medication non-adherence [13]. An intervention study was performed in community pharmacies in the Netherlands identifying and assessing patients who were non-adherent to cardiovascular medication by using the QBS. In this study, an intervention was tailored to the individual needs of the patient by means of a consultation with the pharmacist [25].

In this randomised controlled trial, we will build on the results from these prior studies. In order to identify a participant’s non-adherence profile(s), an adapted version of the QBS will be used. Two adaptations were made to develop the Adapted QBS questionnaire, namely (I) it was redesigned to be used a questionnaire instead of a semi-structured interview guide and (II) questions that functioned as a screener for depression were removed and replaced with questions that evaluate well-being [26–28]. As a result, the Adapted QBS questionnaire consists of sixteen questions. This questionnaire will be used to classify participants in one or more of the four pre-defined non-adherence profiles, namely (I) Knowledge and perceptions, (II) Practical problems, (III) Side effects, and (IV) Negative mood and beliefs. Additional files includes the adapted QBS and the classification into the non-adherence profiles.

#### Interventions

The four non-adherence profiles correspond to a pre-defined set of supporting modules as indicated in Table 1. All of the supporting modules are theoretically grounded and/or have previously been shown to improve medication non-adherence. A more detailed description of the supporting modules is presented in Additional files.

**Table 1:** Supporting modules based on the non-adherence profiles

Non-adherence profile	Supporting modules
**I. Knowledge and perceptions**	Brief messaging Clinical medication review
**II. Practical problems**	Clinical medication review Medication schedule Reminding messaging* Medication dispensing systems Smart messaging*
**III. Side effects**	Clinical medication review Referral to general practitioner
**IV. Negative mood and beliefs**	Brief messaging Clinical medication review Unguided web-based self-help application for low mood

*Smart messaging and reminding messaging not combined

### Intervention description {11a}

#### Intervention group

The personalised intervention programme will be carried out in three steps. Firstly, the participants are automatically profiled into one or more of the four non-adherence profiles after they have filled in the Adapted QBS questionnaire. The pharmacist receives the Adapted QBS questionnaire, a participant’s non-adherence profile(s) and adjoining supporting modules via the data management platform Castor EDC [29]. Secondly, participants will have an in person, telephone call or video call appointment with the trained pharmacist for shared decision making on the personalised intervention programme. This shared decision making will be based on the participant’s non-adherence profile(s). The appointment with the pharmacist will last approximately 30 min, but this can vary depending on the problems that are identified. Thirdly, the personalised intervention programme will be carried out for a period of 6 months. Supporting modules will be delivered via the mobile phone of the participant (i.e. brief messaging, reminding messaging, smart messaging and unguided web-based self-help application for low mood with the last one also accessible via tablet or computer) and by the pharmacist (i.e. medication schedule, clinical medication review, medication dispensing systems and referral to the general practitioner (GP)). Additional files presents a more detailed description of the supporting modules.

#### Control group

Participants that are assigned to the control group will also fill in the Adapted QBS questionnaire, but will receive usual care including access to a publicly available informative diabetes website. In the Netherlands, participants will be advised to access the ‘Diabetes Foundation website’ [30] and in the UK the ‘Diabetes UK website’ [31]. These websites include information about diabetes, medication, complications and diet. Participants can visit the website as often as they want but will not receive reminders to visit the website. Usual care will be provided by the pharmacist and GP according to the national health care standards. Participants in the control group will also have an in person, telephone call or video call appointment with the pharmacy at baseline in which the participant is informed about the diabetes website. This appointment will however be with the pharmacy assistant instead of the pharmacist in order to avoid delivery of the intervention.

### Criteria for discontinuing or modifying allocated interventions {11b}

One month after the personalised intervention programme was initiated, participants in the intervention group will be contacted by telephone by a member of the research team for a semi-structured interview. This interview will focus on the satisfaction participants have with the selected supporting modules. After this telephone call, a member of the research team will be able to moderate the intensity of some of the interventions (e.g. reduce the frequency of brief messaging) or can actively refer to the pharmacist for other adaptations of the programme (e.g. add a medication box).

### Strategies to improve adherence to interventions {11c}

It is expected that personalisation of the intervention programme for participants in the intervention group will function as a strategy to improve adherence to the programme.

#### Pharmacy team training

All participating pharmacy team members, i.e. pharmacists and pharmacy assistants, will be trained by means of training videos providing information about the study procedures. The training for pharmacists will focus on intervention delivery and for the pharmacy assistants on delivery of the control condition. Each pharmacy site will also be provided with a study protocol including detailed information about the study procedures. Pharmacists and pharmacy assistants will be supported through the data management platform Castor EDC that systematically guides them in the delivery of either the intervention or control condition.

### Relevant concomitant care permitted or prohibited during the trial {11d}

There are no restrictions regarding concomitant care during the study.

### Provisions for post-trial care {30}

There are no provisions for post-trial care after the study has ended.

### Outcomes {12}

#### Primary outcome measure

The primary outcome measure of the study is medication adherence assessed with a telephone pill count that is carried out based on a previously validated approach (Langendoen-Gort M, Rutters F, Huijts D, Elders PJM, Terwee CB, Hugtenburg JG: Validation of an announced telephone pill count compared to a home-visit pill count in people with type 2 diabetes or cardiovascular disease, Submitted). A member of the research team will conduct the telephone pill count with the use of a standardised protocol. The participant will be asked to gather all of their medication and count the number of remaining pills from their oral antidiabetic and/or antihypertensive medication. Calculation of the pill count will be performed by using the following equation: (number of dosage units dispensed − number of dosage units remaining) / (prescribed number of dosage units per day × number of days between two telephone pill count 1 and count 2) [32].

#### Secondary outcome measures

The secondary outcome measures of the study are systolic blood pressure, HbA1c level, self-reported medication adherence, attitude and beliefs toward medication, satisfaction with diabetes treatment, health status and medical consumption and productivity cost. An overview of all the assessments is presented in the Standard Protocol Items Recommendations for Interventional Trials (SPIRIT) diagram in Table 2.

**Table 2:** SPIRIT diagram (*t*_0_= baseline; *t*_1_=1 month after baseline; *t*_2_=3 months after baseline; *t*_3_=6 months after baseline) [33]. ^*^Satisfaction phone call will only be performed in participants that receive the intervention condition

	Study period						
	Enrolment	Pre-randomisation	Randomisation	Post-randomisation			
**TIME POINT**				t_0_	t_1_	t_2_	t_3_
**ENROLMENT**							
In-practice electronic search	X						
Written invitation	X						
Information phone call, including eligibility screening	X						
Informed consent	X						
Randomisation			X				
**INTERVENTIONS**							
Intervention							
Control							
**ASSESSMENTS**							
Telephone pill count				X			X
Blood pressure				X			X
HbA1c				X			X
Satisfaction phone call*					X		
Adapted QBS		X					
Demographics		X					
MARS-5		X				X	X
BMQ Specific		X				X	X
DTSQs		X				X	X
DTSQc							X
EQ-5D-5L		X				X	X
iMTA costs						X	X

##### Systolic blood pressure and HbA1c level

To assess a participant’s systolic blood pressure and HbA1c level at baseline, general practice registry data will be consulted with a time window of 3 months before to 1 month after the time point. At the end of the follow-up, general practice registry data will also be consulted, but with a time window of 2 months before to 2 months after the time point. Data will be collected through a telephone call conducted by a member of the research team.

##### Self-reported medication adherence

To assess a participant’s self-reported medication adherence the Medication Adherence Report Scale (MARS-5) will be used at baseline and after 3 and 6 months. The MARS-5 questionnaire consists of five items that enable the researcher to distinguish between intentional and unintentional non-adherence. Four items are focused on intentional non-adherence (e.g. “I take less than instructed) and one item is focused on unintentional non-adherence (i.e. “I forget to take it”). All items are scored on a 5-point scale (i.e. always, often, sometimes, rarely, never). The internal reliability (Cronbach’s *α*) of the MARS-5 ranged from 0.67 to 0.89 in different patient populations, with a Cronbach’s *α* of 0.89 in people with diabetes, and a test-retest reliability (Pearson’s *r*) of 0.97 in people with hypertension [34].

##### Attitude and beliefs toward medication

To assess a participant’s attitude and beliefs toward medication, the Beliefs about Medicines Questionnaire Specific (BMQ Specific) will be used at baseline and after 3 and 6 months. The BMQ Specific questionnaire consists of two subscales, namely the Specific-Necessity and Specific-Concerns. The BMQ Specific-Necessity measures a participant’s beliefs about the necessity of taking medication in order to maintain their health and the BMQ Specific-Concerns assesses a participant’s concerns about taking medication. The BMQ Specific questionnaire consists of ten items that are all scored on a 5-point Likert scale (i.e. strongly agree, agree, uncertain, disagree, strongly disagree). Both the Specific-Necessity and Specific-Concerns consist of five items. The internal consistency (Cronbach’s *α*) of the Specific-Necessity ranged from 0.55 to 0.86, with a Cronbach’s *α* of 0.74 in people with diabetes. The internal consistency (Cronbach’s *α*) of the Specific-Concerns ranged from 0.63 to 0.80, with a Cronbach’s *α* of 0.80 in people with diabetes. Test-retest reliability was 0.77 and 0.76 for respectively the Specific-Necessity and Specific-Concerns in asthmatic patients [35].

##### Satisfaction with diabetes treatment

To assess a participant’s satisfaction with their diabetes treatment, the Diabetes Treatment Satisfaction Questionnaire (DTSQ) will be used at baseline and after 3 and 6 months. The DTSQ consists of two versions, namely the status (DTSQs) and change (DTSQc) version. The DTSQs will be used at baseline and after 3 and 6 months to assess a participant’s status score. The DTSQc will be used after 6 months to assess how a participant’s treatment satisfaction has changed and accounts for possible ceiling effects [36]. Both the DTSQs and DTSQc questionnaires consist of eight items that are all scored on a seven-item answer scale [36]. The internal reliability (Cronbach’s *α*) of the DTSQs ranged from 0.70 to 0.88 [37].

##### Health status

To assess a participant’s health status the EuroQol five-level (EQ-5D-5L) questionnaire will be used at baseline and after 3 and 6 months. The EQ-5D-5L includes statements on five health dimensions (i.e. mobility, self-care, usual activities, pain/discomfort, anxiety/depression) that are scored on five-item severity scales (i.e. no problems, slight problems, moderate problems, severe problems, unable to/extreme problems) [38]. Results on the EQ-5D-5L health states will be used to develop utility scores. These utility scores will be used to calculate quality-adjusted life years (QALYs). In addition, the EQ-5D-5L consists of a visual analogue scale with endpoints ranging from 0 and 100 at which participants indicate their current perceived health state [38].

#### Costs

To assess a participant’s medical consumption and productivity cost items from the iMedical Consumption Questionnaire (iMCQ) and iProductivity Cost Questionnaire (iPCQ) will be used after 3 and 6 months. These questionnaires were targeted to our study population of people with T2DM. By using both the iMCQ and iPCQ, costs will be measured from a societal perspective, considering health care use, homecare, work presenteeism and work absenteeism [39, 40]. A participant’s prescribed medication is obtained from the pharmacy information system.

### Participant time line {13}

**Fig. 1:**
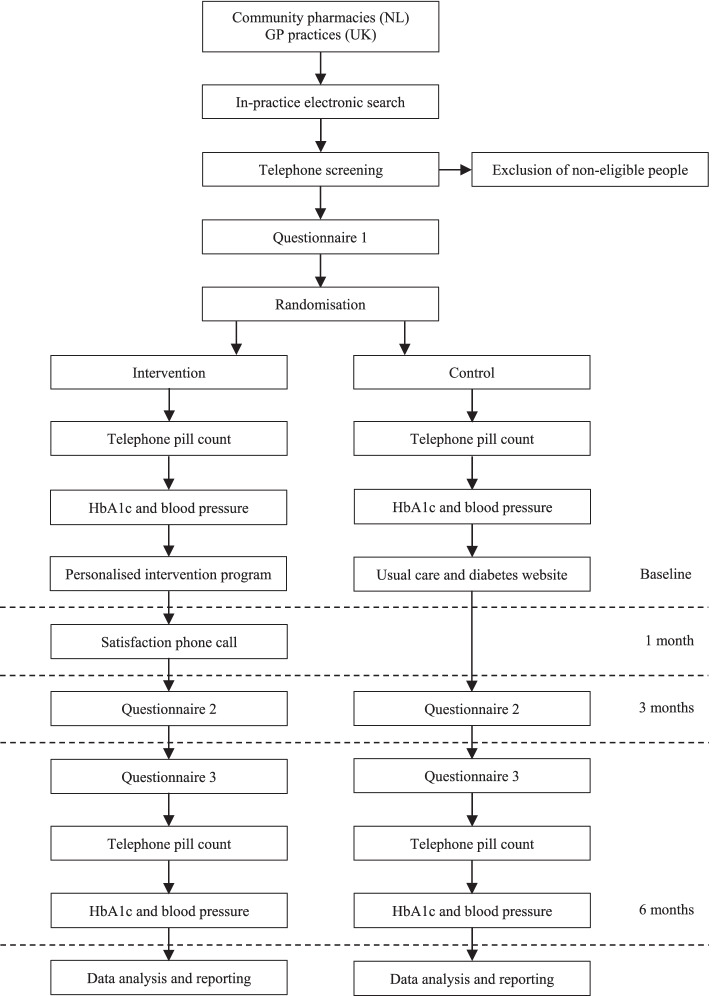
Schematic overview of the study design of the INTENSE study

When participants are enrolled in the study (i.e. after the informed consent procedure), they will fill in the first questionnaire (i.e. Adapted QBS, Demographics, MARS-5, BMQ Specific, DTSQs and EQ-5D-5L). After the first questionnaire, participants will be randomised to either the intervention or control group. Following randomisation, telephone pill counts will be conducted and blood pressure and HbA1c values will be obtained from registry data. One month after the personalised intervention programme was initiated, a satisfaction phone call will be conducted with participants that are randomised to the intervention group. Three and 6 months after study start, participants respectively will fill in the second (i.e. MARS-5, BMQ Specific, DTSQs, EQ-5D-5L and iMTA costs) and third questionnaires (i.e. MARS-5, BMQ Specific, DTSQs, DTSQc, EQ-5D-5L and iMTA costs). Table 2 provides an overview of all the assessments according to the SPIRIT, and Fig. 1 presents a schematic overview of the study design of the INTENSE study.

### Sample size {14}

The sample size calculation was based on multilevel analysis with the pharmacy, patient and time as different levels and we assumed a relative difference of 20% in medication adherence between the intervention and control group [41]. The relative difference was based on what the study team deemed to be clinically relevant. Both in the intervention and control groups, 98 participants are needed, considering a type I error rate of 5% and power of 0.80. In addition, taking the cluster design of the study into account, and assuming an intra-correlation coefficient of 0.10, a total of 110 participants are needed in both trial arms. To adjust for an expected loss to follow-up of 25%, we will need to include 145 participants in both the intervention and control group. In total, 300 participants will be included, namely 150 in the Netherlands and 150 in the UK. Additional power calculations for the secondary outcomes showed that this would also be sufficient to detect a difference of 0.5% in HbA1c and 5 mmHg in systolic blood pressure.

### Recruitment {15}

#### Pharmacy and general practice recruitment

In the Netherlands, pharmacies will be recruited throughout the country. Pharmacies will be provided with an information sheet via email entailing details about the study. When pharmacies express interest in participation in the study, a member of the research team will contact them via telephone and provides them with additional information after which pharmacies can decide if they want to participate in the study. Participating pharmacies will receive a voucher of €25 for every participant they guide during the study. General practices that will be consulted for registry data on participants’ HbA1c and blood pressure levels will not actively be recruited since they are participants’ caregivers and will receive a voucher of €15 for every participant.

In the UK, remuneration of pharmacies and medical practices will be in accordance with nationally agreed UK tariffs for research. Calculations will include remuneration for medical practices to screen and invite eligible patients and provide baseline and follow-up clinical data for participants. The approximate remuneration will be £500 plus support costs provided by the national clinical research network. Community pharmacies will be remunerated for undertaking training and setting up the site for recruitment, consenting patients, collecting data and intervention delivery. The approximate remuneration will be £100 per patient with an estimated 30 participants per pharmacy.

#### Participant recruitment

As previously described, participants will be selected with an in-practice electronic search in the dispensing records of participating pharmacies by pharmacists in the Netherlands and in the prescribing records of participating general practices by a member of the general practice team in the UK. In the Netherlands, potentially eligible participants are provided with an invitation and information brochure via post as send out by pharmacies. In the UK, invitation and information is provided by general practice teams. Potential participants will express their interest by contacting the researchers in the Netherlands and in the UK by contacting the pharmacies. After expressing interest, informed consent is obtained by a member of the research team. Participating participants will receive a voucher of €10 for participation in the Netherlands and £8.50 in the UK to cover out of pocket expenses arising from study-related internet costs.

## Assignment of interventions: allocation

### Sequence generation {16a}

All participants that provide informed consent and that are eligible for the study will be randomised by variable block randomisation, with a blinded block size of 2:4. The randomisation will be stratified per pharmacy, conducted at participant level and will be normalised for age using a group of 35–55 years and 56–75 years. The sequence generation is performed by the data management platform Castor EDC [29].

### Concealment mechanism {16b}

The randomisation sequence is concealed to those who enrol the patients for the study.

### Implementation {16c}

As soon as a member of the research team has indicated in the data management platform Castor EDC that the participant is suitable for the study and that the participant has given informed consent, allocation will be indicated by Castor EDC [29].

## Assignment of interventions: blinding

### Who will be blinded {17a}

Blinding of the research team, pharmacy team, participants and GPs to treatment allocation is not possible due to the nature of the study.

### Procedure for unblinding if needed {17b}

Because the study is not blinded, a procedure for unblinding is not applicable.

## Data collection and management

### Plans for assessment and collections of outcomes {18a}

Both the research team and pharmacy team will be able to log into the data management platform Castor EDC and introduce the data directly into the system. For some quantitative data, the platform performs a range check and prompts if data are out of range. All questionnaires will be sent automatically as electronic forms to the participants and filled in directly into the data management system. If participants indicate not being able to handle the electronic forms, paper questionnaires will be sent and returned by post and answers will be introduced into the data management platform by a member of the research team. This will be done with a single data introduction procedure that is often combined with a prompt to check whether the introduced data are correct.

The Evalan platform will automatically send out the DTSQs and DTSQc questionnaires as electronic forms to participants and will be used to carry out some of the supporting modules (i.e. brief messaging, reminding messaging and smart messaging).

### Plans to promote participant retention and complete follow-up {18b}

Participants will be reminded twice by the data management platform Castor EDC and Evalan platform in case of electronic questionnaires or by a member of the research team in case of paper questionnaires if no reply has been received. In case of electronic questionnaires, this will be followed by a telephone call conducted by a member of the research team.

### Data management {19}

Both contributing countries will have a separate Castor EDC data management and Evalan platform in which all data will be stored except participant identifiable data that will be stored separately due to aspects of confidentiality.

### Confidentiality {27}

Pharmacies will only be able to access the data that they enter and view the information required to deliver the interventions. The research team in each country will have a log in code to view all data of each participant and will be able to enter data resulting from the telephone calls and paper questionnaires. Apart from the email address of the participant that is needed for sending out the questionnaires, participant identifiable data and audiotapes (see also interim analysis {21} and additional analysis {20}) will be stored separately under lock in each country. These will be only accessible by the country-specific research team. During the study, the research team will have limited access to the management system of each country and the anonymised transcriptions of the interviews of each country. After study completion, all research data collected in the UK will be pseudonymised and transferred to the Netherlands, where it will be stored alongside the research data from the Netherlands for 15 years. Participant identifiable information collected in the UK will be stored in the UK for 12 months after the end of the study.

### Plans for collection, laboratory evaluations and storage of biological specimens for genetic or molecular analysis in this trial/future use {33}

Not applicable, because biological specimens will not be collected or stored.

## Statistical methods

### Statistical methods for primary and secondary outcomes {20a}

#### Effectiveness analysis

Descriptive statistics will be used to describe the study population. Missing data will be imputed by multiple imputation. The effect analyses will be performed according to an intention-to-treat principle, and differences in the primary and secondary outcome measures between the intervention and control group will be analysed with multilevel regression analysis. We shall assess whether age, even after normalising randomisation for this variable, is an interaction factor with regard to the outcome of this study. A *p*-value of <0.05 will be considered statistically significant.

#### Cost-effectiveness analysis

The cost-effectiveness analysis will be performed from a societal perspective. Missing data will be imputed by multiple imputation. The imputed datasets will then be pooled using Rubin’s rules. Incremental cost-effectiveness ratios (ICERs) will be calculated by dividing the difference in mean costs between the intervention and control group by the difference in mean effects between the two groups. Bootstrapping with 5000 replications will be used to estimate bias-corrected and accelerated 95% confidence intervals around cost differences. The bootstrapped incremental cost-effect pairs will be plotted on cost-effectiveness planes, illustrating uncertainty surrounding the ICERs. Cost-effectiveness acceptability curves will also be presented to show the probability that the personalised intervention programme is cost-effective in comparison with the control group for a range of different ceiling ratios.

#### Process evaluation

A process evaluation assesses the extent to which the personalised intervention programme has been performed according to the study protocol and gives insight into the barriers and facilitators in the execution of the intervention programme. We shall assess fidelity, dose and reach according to the Medical Research Council (MRC) process evaluation framework of complex interventions [42, 43]. We will also use the Extended Normalisation Process Theory as a theoretical framework, in order to focus on the feasibility of the intervention programme to be normalised in daily practice [44]. We will use a mixed-methods approach incorporating activities recorded by pharmacists into the Castor EDC data management platform, audio recordings of a sample of pharmacist shared decision making consultations and interviews with relevant stakeholders. These interviews will be conducted among a purposive sample of nine stakeholders involved in the personalised intervention programme, namely participants, pharmacists and in the UK, members of the general practice team. Interviews will be conducted during the pilot phase and overall trial. Interview data will be combined with data recorded in the online data management platform.

The fidelity framework will capture whether the pharmacist conducted shared decision making with the participant on supporting modules to use and whether the pharmacist initiated the supporting modules (i.e. clinical medication review, medication schedule, medication dispensing systems and referral to GP) correctly. These data will be captured from the Castor EDC data management platform and stakeholder interviews. Audio recordings of pharmacists’ consultations will further determine the quality of consultation skills and pharmacists’ compliance to consultation delivery. Through qualitative analysis of participant interviews, we will gain the perspective and opinion of participants on the content of the intervention received. We shall assess through the interviews whether participants and pharmacists thought that the right participants were selected for the study.

The Extended Normalisation Process Theory focusses on the effort that stakeholders have to put into an intervention in order for a complex intervention to normalise and consists of four core elements, namely (I) potential, (II) capacity, (III) capability and (IV) contribution [44]. This evaluation will be based on the interviews with pharmacists.

Table 3 provides an overview of the components of the MRC process evaluation framework of complex interventions and the Extended Normalisation Process Theory.

**Table 3:** Framework to perform the process analysis with components of the MRC process evaluation framework of complex interventions [42, 43] and the Extended Normalisation Process Theory [44]. ^*^ HCP refers to pharmacists and/or members of the general practice team

Theory	Theme	Description	Source
**The MRC process evaluation framework of complex interventions**	Fidelity	Delivery of intervention as intended Personalised intervention programme delivered	Interviews with participants and HCPs* Audio recordings from the consultations Entries made by the pharmacist in the data management platform Satisfaction phone call and data recorded in the data management platform after 1 month
	Dose delivered	Personalised intervention programme delivered	Interviews with participants and HCPs* Audio recordings from the consultations Satisfaction phone call and data recorded in the data management platform after 1 month
	Dose received	Intervention received and participants engagement with the intervention	Interviews with participants and HCPs* Quantitative data from practices and pharmacies
	Reach		Interviews with participants and HCPs* Quantitative data from practices and pharmacies
	Recruitment/context	Reasons to participate Barriers to participate	Interviews with participants and HCPs*
**Extended Normalisation Process Theory**	Potential	Commitment Assessment of change efficacy	Interviews with HCPs*
	Capability	Experiences with the intervention Workability	
	Capacity	Changes needed in working processes Feasibility of this change	
	Contribution	Distribution of tasks	

### Interim analysis {21b}

An interim process evaluation analysis will be performed after we have included approximately 25 participants per country. The aim of this analysis is to test the logistics, measurements, questionnaires, training of pharmacists and the usability of other study materials. One month after the start of the study and throughout the study, semi-structured interviews will be conducted among a purposive sample of relevant stakeholders, participants and pharmacists, to conduct a process evaluation. We expect to perform nine interviews in each country. Based on the outcomes of the process evaluation, potential adjustments will be made to the study design during the entire study. With regard to effectiveness of the personalised intervention programme, no interim analysis will be performed.

### Methods for additional analyses (e.g. subgroup analyses) {20b}

#### Non-adherence algorithm

Participants will initially be categorised according to the pre-defined set of non-adherence profiles, namely (I) Knowledge and perceptions, (II) Practical problems, (III) Side effects and (IV) Negative mood and beliefs. After conducting the trial, all available data, including the pre-trial data and log-data, will be used to identify additional factors that influence profiling and profiles will be using subgroup discovery algorithms.

### Methods in analysis to handle protocol non-adherence and any statistical methods to handle missing data {20c}

We shall study protocol non-adherence during the process analysis (Table 3) and will impute missing data by multiple imputation.

### Plans to give access to the full protocol, participant-level data and statistical code {31c}

The protocol and statistical codes can be supplied upon reasonable request to the corresponding author. Participant-level data cannot be supplied because no informed consent will be obtained for data sharing.

## Oversight and monitoring

### Composition of the coordinating centre and trial steering committee {5d}

The coordinating study centre is the Department of General Practice, Amsterdam University Medical Centres, location VUmc, the Netherlands. The Trial Management Committee (TMC) consists of members of the Amsterdam University Medical Centres, Oxford University and University of East Anglia and will be responsible for all aspects of the study planning, randomisation, data registration, data management, biostatistics and study monitoring. The coordinating study centre organises the TMC meetings which are held on average once every 2 months. In addition, in the UK an advisory group has been established. This group comprises of six independent members (i.e. a chair, statistician, behavioural scientist and three patient representatives) and three non-independent members from the TMC. They will convene during the internal pilot phase and thereafter at critical time points.

### Composition of the data monitoring committee, its role and reporting structure {21a}

In the Netherlands, the study will be monitored by a monitor from the Clinical Research Bureau, Amsterdam University Medical Centres, the Netherlands. The University of East Anglia (UK sponsor) decided that no data monitoring board was needed due to the fact that the study was deemed to be in the lowest risk category.

### Adverse event reporting and harms {22}

All adverse events spontaneously reported by participants, the pharmacy team, GP’s or observed by the research team will be recorded.

### Frequency and plans for auditing trial conduct {23}

The study in the Netherlands will be monitored by a monitor from the Clinical Research Bureau, Amsterdam University Medical Centres, the Netherlands. Monitoring will take place at the coordinating study centre and a sample of participating pharmacies. The coordinating study centre will undergo at least two monitor visits during the trial and a closing visit at the end of the trial. The pharmacies will at least undergo one monitor visit.

### Plans for communicating important protocol amendments to relevant parties (e.g. trial participants, ethical committees) {25}

Substantial amendments will be communicated to the ethical committee and reported in follow-up publications.

### Dissemination plans {31a}

We aim to publish the process evaluation of the trial and the (cost-)effectiveness study separately in peer-reviewed international papers. We will also prepare materials to share key messages with health care professionals and the general public.

## Discussion

This paper describes the background and study design of the INTENSE study, a study to evaluate the (cost-)effectiveness of a personalised intervention programme compared to usual care in people with T2DM that are non-adherent to oral antidiabetic and/or antihypertensive medication. This personalised intervention programme is hypothesised to increase medication adherence by identifying an individual’s barriers to medication adherence and providing intervention modules to overcome these barriers. We hypothesise that improved medication adherence will lead to a decrease in HbA1c levels and systolic blood pressure and as a result decrease the risk of long-term diabetes complications and health care costs and improve health status of people with T2DM.

This complex multidisciplinary study brings about a number of challenges. The first challenge is the use of a telephone-based pill count as the primary outcome of this study, which is not a widely used measure for medication adherence [45–50]. However, there is currently no gold standard available for the measurement of medication adherence and, therefore, researchers should assess which medication adherence measure is most suitable for their study [32]. We selected a telephone-based pill count since it is practical, has low implementation costs [46, 48] and showed high concordance with home-based pill counts that are conducted more extensively in other patient populations [46–48, 50]. Also, we performed a validation of a telephone-based pill count in 34 people with T2DM or cardiovascular disease accounting for a total of 203 pill counts. In this validation study, a high concordance between a telephone-based and home-based pill count was observed with an intraclass correlation coefficient (ICC) of 0.96 (95% CI 0.94–0.97) (Langendoen-Gort M, Rutters F, Huijts D, Elders PJM, Terwee CB, Hugtenburg JG: Validation of an announced telephone pill count compared to a home-visit pill count in people with type 2 diabetes or cardiovascular disease, Submitted).

The second challenge is that participants will be randomised at individual level, leading to one pharmacy treating both participants in the intervention and control group. This randomisation procedure could potentially induce contamination between the intervention and control group. In order to reduce this contamination risk, the intervention condition will be carried out by the pharmacist and the control condition will be carried out by the pharmacy assistant. Also, pharmacy assistants will not be able to view a participant’s answers on the Adapted QBS questionnaire if they are randomised to the control group.

A final challenge is the identification of people that are truly non-adherent by conducting an in-practice electronic search in the dispensing records. One of the explanations that previously developed adherence interventions were proven ineffective was that they were offered to all patients regardless of whether they were non-adherent [12, 13]. We have accounted for this by excluding people that have invalid adherence data due to extensive hospital admissions or that have their medication dispensed by multiple pharmacies, inducing gaps in the dispensing records. However, participants that use the pharmacies’ repeat dispensing service and frequently pick up their medication could appear adherent in the dispensing records, also if medication is not taken as prescribed. Therefore, the use of an automated search in dispensing records to identify participants can result in a group of non-adherent people with T2DM that are not identified as non-adherent and as such are not offered the intervention. Unfortunately, this is inherent to this type of research and could potentially induce sample selection bias.

Moreover, the study benefits from a number of strengths. The first strength is the personalised intervention programme that is tailored to the individual needs of participants. One of the explanations that previously developed adherence interventions were proven ineffective was that they were not tailored to the individual needs of participants [12, 17]. Personalisation of our intervention programme will be attained by several components. Individual barriers for medication adherence will be identified with the Adapted QBS questionnaire. Moreover, the participant will have an appointment with their pharmacist to discuss these barriers and for shared decision making on the supporting modules to be initiated. Finally, 1 month after the start of the study the participant and researcher will evaluate the personalised intervention programme and adjustments can be made to an individuals’ programme if this is deemed necessary.

A second strength of the study is that the randomised controlled trial will be performed in both the Netherlands and the UK, improving the generalisability of the study results. However, alignment of the study protocol to the different healthcare settings in the Netherlands and the UK has been challenging. An example of such a challenge is that in the Netherlands, prescription data is available at the pharmacy whereas in the UK this data is available at the general practice. However, by bridging these differences, we have been able to develop an intervention that should be usable in both the Dutch and UK healthcare systems. Moreover, it shows the possibilities that the intervention can be scalable to other European countries.

A final strength of the study is that alongside the clinical trial, a process evaluation will be performed. This process evaluation will provide insight into the fidelity and reach of the programme and the barriers and facilitators in the execution of the intervention programme. Research shows that there is increasing interest in the evaluation of complex interventions and it is recommended that this evaluation is guided by a theoretical framework [51]. For this process evaluation, we shall assess fidelity, dose and reach according to the MRC process evaluation framework of complex interventions and the Extended Normalisation Process Theory as a theoretical framework, in order to focus on the feasibility of the intervention programme to be normalised in daily practice [44]. Results of this process evaluation can be highly relevant for other researchers developing medication adherence interventions and for policy makers that are involved in the implementation of these interventions.

If this study shows that the INTENSE intervention is (cost-)effective, it can be considered for use in other patient populations since medication non-adherence is also frequently reported among other patient groups. Moreover, extrapolation to other European countries can be considered.

## Trial status

The pilot study started in October/November 2020 but was periodically paused due to the COVID-19 Pandemic. Inclusion of participants was ongoing during manuscript submission. Recruitment will end July 31, 2022. Study results are expected at the end of 2022.

## Supplementary Information

**Additional file 1.** The Adapted QBS questionnaire and the classification into the non-adherence profiles.

**Additional file 2.** A description of the supporting modules.

## Data Availability

The protocol and statistical codes can be supplied upon reasonable request to the corresponding author. Participant-level data cannot be supplied because no informed consent will be obtained for data sharing.

## Abbreviations

BMQ Specific: Beliefs about Medicines Questionnaire Specific
DTSQ: Diabetes Treatment Satisfaction Questionnaire
DTSQc: Diabetes Treatment Satisfaction Questionnaire Change
DTSQs: Diabetes Treatment Satisfaction Questionnaire Status
EFSD: European Foundation for the Study of Diabetes
EQ-5D-5L: EuroQol five-level
GP: General Practitioner
ICERs: Incremental cost-effectiveness ratios
iMCQ: iMedical Consumption Questionnaire
INTENSE: ImproviNg Treatment adhErence iN people with diabeteS mEllitus
iPCQ: iProductivity Cost Questionnaire
MARS-5: Medication Adherence Report Scale
MRC: Medical Research Council
QALYs: Quality-adjusted life years
QBS: Quick Barrier Scan
SPIRIT: Standard Protocol Items Recommendations for Interventional Trials
T2DM: Type 2 diabetes mellitus
TMC: Trial Management Committee
UK: United Kingdom
