# Supplementary material for: A personalised intervention programme aimed at improving adherence to oral antidiabetic and/or antihypertensive medication in people with type 2 diabetes mellitus, the INTENSE study: study protocol for a randomised controlled trial
Source: Trials. 2022 Sep 2;23:731. doi: 10.1186/s13063-022-06491-7 (PMC9438235; doi:10.1186/s13063-022-06491-7)
Supplement: Supplementary file 1 — Additional file 1. The Adapted QBS questionnaire and the classification into the non-adherence profiles. [file 13063_2022_6491_MOESM1_ESM.pdf]

ADDITIONAL FILE 1

Table A1. The Adapted QBS questionnaire and the classification into the non-adherence profiles (part 1) (①= Knowledge and perceptions; ②= Practical problems; ③= Side effects; ④= Negative mood and beliefs)

Statement	Yes	No	Non-adherence profile number (in case 'yes' was answered)
1. Do you feel that you have insufficient knowledge about your diabetes or medicines?			①
2. Do you ever forget to take your medicines on regular days?			②
3. Do you ever forget to take your medicines on irregular days, for example when you are away for the weekend or on vacation?			②
4. Do you experience side effects of your medicines?			③
5. Are you sometimes worried about getting side effects from your medicines?			③
6. Do you ever experience difficulties with the amount of medicines you have to take or the different intake moments?			②
7. Do you ever experience difficulties with opening packages or swallowing pills?			②
8. Do you ever worry about taking medicines in general? Do you for instance think that doctors use too many medicines, that medicines do more harm than good and/or that medicines have a bad influence on the body?			④
9. Do you ever feel like you do not need your medicines?			④
10. Do you ever feel like your medicines are not effective or have			④

more disadvantages than advantages?			
11. Do you have any other ideas why it could be difficult for you to take your medicines?<br><br>When your answer to this statement is yes, please indicate briefly what these ideas are:<br>………………..............			①<br>②<br>③<br>④

Table A2. The Adapted QBS questionnaire and the classification into the non-adherence profiles (part 2) (1-3) (Calculation of statements 12 to 16: sum score * 4 = total score. A total score of 29-50 indicates non-adherence profile ④)

**Statement**	**All of the time**	**Most of the time**	**More than half of the time**	**Less than half of the time**	**Some of the time**	**At no time**
**Score**	**5**	**4**	**3**	**2**	**1**	**0**
12. I have felt cheerful and in good spirits						
13. I have felt calm and relaxed						
14. I have felt active and vigorous						
15. I woke up feeling fresh and rested						
16. My daily life has been filled with things that interest me						

Table A3. Supporting modules based on the non-adherence profiles * Smart messaging and reminding messaging not combined

NON-ADHERENCE PROFILE	SUPPORTING MODULES
① **Knowledge and perceptions**	Brief messaging<br>Clinical medication review
② **Practical problems**	Clinical medication review<br>Medication schedule<br>Reminding messaging*<br>Medication dispensing systems<br>Smart messaging*
③ **Side effects**	Clinical medication review<br>Referral to general practitioner
④ **Negative mood and beliefs**	Brief messaging<br>Clinical medication review<br>Unguided web-based Self Help Application for low mood
