# Supplementary material for: A personalised intervention programme aimed at improving adherence to oral antidiabetic and/or antihypertensive medication in people with type 2 diabetes mellitus, the INTENSE study: study protocol for a randomised controlled trial
Source: Trials. 2022 Sep 2;23:731. doi: 10.1186/s13063-022-06491-7 (PMC9438235; doi:10.1186/s13063-022-06491-7)
Supplement: Supplementary file 2 — Additional file 2. A description of the supporting modules. [file 13063_2022_6491_MOESM2_ESM.pdf]

ADDITIONAL FILE 2

DESCRIPTION OF THE SUPPORTING MODULES

Based on an individuals' answers provided to the Adapted QBS questionnaire and consequent classification into one or more of the non-adherence profile(s) (see **Table 1** and **Additional file 1**), the pharmacist discusses the optional supporting modules. **Table A4** provides a brief description of the different supporting modules.

Table A4. Brief description of the supporting modules

SUPPORTING MODULE	DELIVERY	BACKGROUND INFORMATION AND DESCRIPTION OF SUPPORTING MODULE
**Brief messaging**	Evalan platform(4)	It has been shown that the use of brief SMS messages can result in an improvement of medication adherence in people with T2DM(5, 6) and in people using high blood pressure medication(7).<br>For the brief messaging module, brief educational messages with a fixed length of 160 characters were developed. The messages were developed in three brainstorm sessions with researchers and people working within a GP practice and evaluated by three patient panels in the Netherlands consisting of people with T2DM. The messages were developed within several domains of the Behavioural Change Technique Taxonomy by Michie(8). All messages that were approved by at least one panel were sent to the research team in the UK. After translation they were discussed in a patient panel in the UK and suggestions for change were made by the UK research team. The rephrased UK suggestions were again discussed within the research team of both countries until consensus was reached. In total 48 messages covering, according to the research teams of both countries, 12 domains of the Behavioural Change Technique Taxonomy by Michie et al.(8) were selected and put in a fixed order, so that participants will receive a wide range of informational issues on diabetes and diabetes medication covering the domains of the taxonomy without too much chronological overlap. Participants will receive either one or two text messages per week for a period of six

		months. **Table A5.** provides an indication of the content of the brief messages and their classification into the Behavioural Change Technique Taxonomy by Michie(8).
**Reminding messaging**	Evalan platform(4)	Text messaging including predetermined fixed reminding (reminding messaging) has been shown to increase medication adherence(9, 10).<br><br>Participants will receive reminding text messages. During the consultation the pharmacist and participant decide at which time points the participants would like to receive reminding messages. Participants thereafter receive a reminding message at the time point they have indicated. The English version of the reminder message is: "This is a reminder to take your medicines."
**Smart messaging**	Evalan platform and electronic medication container(4, 11)	Text messaging including real time medication monitoring (smart messaging) has been shown to increase medication adherence(9). In a trial with T2DM patients the average medication adherence of patients who received smart reminding was found to increase from about 60% before the start of the trial to about 80%(12, 13).<br><br>The participant will receive an electronic medication container that is linked to the Evalan platform. This combination of container and platform has been used in previous studies(12, 14) At baseline, the participant and pharmacist include the time points at which the participant should take their medication. In case of smart messaging the electronic medication container will send a signal to the platform if opened. A participant is reminded via a text message if the medication is not taken within the predefined time frame (i.e. the medication container is not opened). Reminders are not send if the medication is taken as prescribed (i.e. the medication container is opened).
**Clinical medication review**	Pharmacist	Medication adherence is related to multiple interrelating factors in several domains(15). Previously we have shown in people who were taking antihypertensive medication that personal beliefs, practical issues and side effects are important factors in medication adherence and that interventions to improve medication adherence should be tailored to the individual patient(16, 17). A clinical medication review by a pharmacist is ideally suited to address issues and to address medicated related problems. Also it has been shown that the most effective interventions to improve medication adherence were delivered face-to-face, by pharmacists, and administered directly to patients(18). This explains that the option of a clinical medication review is offered to the participants in all non-adherence profiles.

		The pharmacist performs a clinical medication review in cooperation with the GP. In the Netherlands, this review will be performed according to a standard process as provided in the 'Multidisciplinary Guidelines for Polypharmacy'(19). In short, the review consists of the following five steps: (I) Pharmacotherapeutic anamnesis, (II) Pharmacotherapeutic analysis, (III) Consultation GP and pharmacist, (IV) Feedback to participant and other care providers, and (V) Follow-up (19). In the UK, this review will be tailored to individual need and guided by the principles of 'A guide to medication review'(20).
**Medication schedule**	Pharmacist	A medication schedule can be provided by the pharmacist, by using the pharmacy administration and information system. A medication schedule includes the (general) time points at which the participant should take their medication and the amount.
**Medication dispensing systems**	Pharmacist	Several studies indicate the potential of pill packaging and the use of a pillbox for improving medication adherence(21, 22). Moreover, the study of Porter et al. showed a clinically significant decrease in systolic blood pressure, by 10 mmHg, in uncontrolled hypertensive patients that use a pillbox and got an instruction on the use(23).<br>Currently, several medication dispensing systems are available within the pharmacy. Examples of such systems are pill packaging, pillboxes and repeat dispensing service. The pharmacist will discuss with the participant which systems are available and provides the option(s) that comply with the needs of the participant.
**Unguided web-based Self Help Application for low mood**	Minddistrict platform(24)	Depressive mood has been found to be related to poor treatment adherence(25, 26). In the past years we have developed a web-based depression intervention for people with diabetes based on Cognitive Behavior Therapy(27) . This guided self-help program ("Betergestemd") proved to be effective in reducing depressive symptoms as well as diabetes distress(28). This program runs on pc's, tablets and smart phones and recently has been further developed to an unguided self-help application ("myDiaMate") which aims to prevent and reduce psychological distress and fatigue in adults with diabetes. The content of "Betergestemd" is incorporated in a specific module on low mood, next to topics on diabetes in balance and fatigue(29). This unguided self-help application was found to be feasible and appreciated by people with diabetes to improve their psychological well-being.<br>In this project we shall offer this unguided version of "Betergestemd" to participants with an indication of low mood based on the WHO-5 questionnaire. The WHO-5 has proven to be a feasible screener for low mood (< 50) and likely

		depression (score ≤ 28)(2). Those who screen positive for likely depression will be prompted to make an appointment with their GP and the GP will be informed by the research group.<br><br>The Unguided web-based Self Help Application for low mood for people with diabetes consists of eight modules. Modules focus on six techniques based on cognitive behavioral therapy: (I) pleasant activity scheduling, (II) cognitive restructuring, (III) relaxation, (IV) communication, (V) coping with worries, and (VI) assertiveness. The modules consist of text and exercises. In addition, participants are asked to keep a mood and relaxation diary. Participants will be advised to complete one module per week.<br><br>For use in the UK the Dutch version of the Unguided web-based Self Help Application for low mood was translated independently by two native English speakers who were living in the Netherlands and fluent in Dutch and discrepancies were discussed and solved. After this, the program was tested, commented on and adapted by a team of patient and public members working with the research team in the UK.
**Referral to a GP**	Pharmacist and GP	There are two cases in which the pharmacist will refer the participant to the GP.<br><br>**1. Side effects**<br>When a participant indicates to experience side effects a clinical medication review will be carried out first. When the side effects cannot be resolved by this medication review the participant will be referred to their GP for a regular consult.<br>**2. Very low mood**<br>We will screen for low mood by using the Adapted QBS. In the Adapted QBS questions of the WHO-5 questionnaire are incorporated. The WHO-5 questionnaire has a score ranging from 0-100. All participants in the intervention group that score ≤50, and that are not receiving any form of psychotherapy, will be offered the Unguided web-based Self Help Application for low mood. All participants, both in the intervention and control group, with a score ≤ 28 will be advised to visit their GP to screen for depression. The GP will be informed of this advice.

Table A5. Brief messages in their fixed sending order sent on a Monday and/or Thursday. The English version of the messages of the first four weeks are shown together with the classification into the domains of the Behavioural Change Taxonomy by Michie et al.(8)

	**MESSAGE**	**DOMAIN**
**Week 1**	Monday: Ask your prescriber or pharmacist to help you make a plan to take your medicines.	Goals and planning
	Thursday: Tell your pharmacist, doctor or nurse about medicines that you have bought over the counter, including herbal products, vitamins and food supplements.	Covert learning
**Week 2**	Monday: Ask your prescriber or pharmacist any questions you have regarding your medicines.	Social support
	Thursday: You might be able to put an alarm on your phone to remind you to take your medicines. Your pharmacist can help you.	Repetition and substitution + Antecedents + Associations
**Week 3**	Monday: Making a list of points to discuss before any appointment is a really good use of your time and means you don't forget anything.	Regulation
	Thursday: Diabetes UK has a great website (https://www.diabetes.org.uk/home) and a helpline (0345 123 2399) which may be of help.	Social support
**Week 4**	Monday: You will experience fewer side effects if you take your medicines regularly.	Consequences + Natural consequences
	Thursday: You are not alone, many people forget to take their medicines.	Shaping knowledge

REFERENCES

1. Bech P. Measuring the dimension of psychological general well-being by the WHO-5. Quality of Life Newsletter. 2004;32:15-6.
2. Hajos TR, Pouwer F, Skovlund SE, Den Oudsten BL, Geelhoed-Duijvestijn PH, Tack CJ, et al. Psychometric and screening properties of the WHO-5 well-being index in adult outpatients with Type 1 or Type 2 diabetes mellitus. Diabet Med. 2013;30(2):e63-9.
3. Topp CW, Ostergaard SD, Sondergaard S, Bech P. The WHO-5 Well-Being Index: a systematic review of the literature. Psychother Psychosom. 2015;84(3):167-76.
4. Evalan. Evalan 2021 [Available from: https://evalan.com/].
5. Arora S, Peters AL, Burner E, Lam CN, Menchine M. Trial to examine text message-based mHealth in emergency department patients with diabetes (TExT-MED): a randomized controlled trial. Ann Emerg Med. 2014;63(6):745-54 e6.
6. Adikusuma W, Qiyaam N. The Effect of Education through Short Message Service (SMS) Messages on Diabetic Patients Adherence. Sci Pharm. 2017;85(2).
7. Leon N, Surender R, Bobrow K, Muller J, Farmer A. Improving treatment adherence for blood pressure lowering via mobile phone SMS-messages in South Africa: a qualitative evaluation of the SMS-text Adherence SuppoRt (StAR) trial. BMC Fam Pract. 2015;16:80.
8. Michie S, Richardson M, Johnston M, Abraham C, Francis J, Hardeman W, et al. The behavior change technique taxonomy (v1) of 93 hierarchically clustered techniques: building an international consensus for the reporting of behavior change interventions. Ann Behav Med. 2013;46(1):81-95.
9. Thakkar J, Kurup R, Laba TL, Santo K, Thiagalingam A, Rodgers A, et al. Mobile Telephone Text Messaging for Medication Adherence in Chronic Disease: A Meta-analysis. JAMA Intern Med. 2016;176(3):340-9.
10. Vervloet M, Linn AJ, van Weert JC, de Bakker DH, Bouvy ML, van Dijk L. The effectiveness of interventions using electronic reminders to improve adherence to chronic medication: a systematic review of the literature. J Am Med Inform Assoc. 2012;19(5):696-704.
11. Evalan. Sensemedic 2021 [Available from: https://sensemedic.com/].
12. Vervloet M, van Dijk L, de Bakker DH, Souverein PC, Santen-Reestman J, van Vlijmen B, et al. Short- and long-term effects of real-time medication monitoring with short message service (SMS) reminders for missed doses on the refill adherence of people with Type 2 diabetes: evidence from a randomized controlled trial. Diabet Med. 2014;31(7):821-8.
13. Vervloet M, van Dijk L, Santen-Reestman J, van Vlijmen B, Bouvy ML, de Bakker DH. Improving medication adherence in diabetes type 2 patients through Real Time Medication Monitoring: a randomised controlled trial to evaluate the effect of monitoring patients' medication use combined with short message service (SMS) reminders. BMC Health Serv Res. 2011;11:5.
14. Vasbinder EC, Goossens LM, Rutten-van Molken MP, de Winter BC, van Dijk L, Vulto AG, et al. e-Monitoring of Asthma Therapy to Improve Compliance in children (e-MATIC): a randomised controlled trial. Eur Respir J. 2016;48(3):758-67.
15. Sabate E. Adherence to long-term therapies: Evidence for action. Geneva: World Health Organization. 2003.
16. van der Laan DM, Elders PJM, Boons C, Nijpels G, Hugtenburg JG. Factors Associated With Nonadherence to Cardiovascular Medications: A Cross-sectional Study. J Cardiovasc Nurs. 2019;34(4):344-52.

17. Hugtenburg JG, Timmers L, Elders PJ, Vervloet M, van Dijk L. Definitions, variants, and causes of nonadherence with medication: a challenge for tailored interventions. Patient Prefer Adherence. 2013;7:675-82.
18. Conn VS, Ruppar TM. Medication adherence outcomes of 771 intervention trials: Systematic review and meta-analysis. Prev Med. 2017;99:269-76.
19. Nederlands Huisartsen Genootschap. Multidisciplinaire Richtlijn Polyfarmacie bij ouderen. 2012.
20. Clyne W, Blenkinsopp A, Seal R. A guide to medication review. National Prescribing Centre; 2008.
21. Conn VS, Ruppar TM, Chan KC, Dunbar-Jacob J, Pepper GA, De Geest S. Packaging interventions to increase medication adherence: systematic review and meta-analysis. Curr Med Res Opin. 2015;31(1):145-60.
22. Schwartz JK. Pillbox use, satisfaction, and effectiveness among persons with chronic health conditions. Assist Technol. 2017;29(4):181-7.
23. Porter AK, Taylor SR, Yabut AH, Al-Achi A. Impact of a pill box clinic to improve systolic blood pressure in veterans with uncontrolled hypertension taking 3 or more antihypertensive medications. J Manag Care Spec Pharm. 2014;20(9):905-11.
24. Minddistrict. Minddistrict 2021 [Available from: https://www.minddistrict.com/].
25. Tiktin M, Celik S, Berard L. Understanding adherence to medications in type 2 diabetes care and clinical trials to overcome barriers: a narrative review. Curr Med Res Opin. 2016;32(2):277-87.
26. Krass I, Schieback P, Dhippayom T. Adherence to diabetes medication: a systematic review. Diabet Med. 2015;32(6):725-37.
27. van Bastelaar KM, Pouwer F, Cuijpers P, Riper H, Snoek FJ. Web-based depression treatment for type 1 and type 2 diabetic patients: a randomized, controlled trial. Diabetes Care. 2011;34(2):320-5.
28. van Bastelaar KM, Pouwer F, Geelhoed-Duijvestijn PH, Tack CJ, Bazelmans E, Beekman AT, et al. Diabetes-specific emotional distress mediates the association between depressive symptoms and glycaemic control in Type 1 and Type 2 diabetes. Diabet Med. 2010;27(7):798-803.
29. Muijs LT, de Wit M, Knoop H, Snoek FJ. Feasibility and user experience of the unguided web-based self-help app 'MyDiaMate' aimed to prevent and reduce psychological distress and fatigue in adults with diabetes. Internet Interv. 2021;25:100414.
